# A Bundle of the “Top 10” Outpatient Parenteral Antimicrobial Therapy Publications in 2023

**DOI:** 10.1093/ofid/ofae635

**Published:** 2024-10-29

**Authors:** Lindsey M Childs-Kean, Alison M Beieler, Nicolás Cortés-Penfield, Sara C Keller, Christina G Rivera, Keenan L Ryan, Leah H Yoke, Monica V Mahoney

**Affiliations:** College of Pharmacy, University of Florida, Gainesville, Florida, USA; Infectious Diseases Clinic, Harborview Medical Center, Seattle, Washington, USA; Division of Infectious Diseases, University of Nebraska Medical Center, Omaha, Nebraska, USA; Division of Infectious Diseases, Department of Medicine, Johns Hopkins University School of Medicine, Baltimore, Maryland, USA; Department of Health Policy and Management, Johns Hopkins Bloomberg School of Public Health, Baltimore, Maryland, USA; Department of Pharmacy, Mayo Clinic, Rochester, Minnesota, USA; Department of Pharmacy, University of New Mexico Hospital, Albuquerque, New Mexico, USA; Vaccine and Infectious Disease Division, Fred Hutch Cancer Center, Seattle, Washington, USA; Allergy and Infectious Disease Division, Department of Medicine, University of Washington School of Medicine, Seattle, Washington, USA; Department of Pharmacy, Beth Israel Deaconess Medical Center, Boston, Massachusetts, USA

**Keywords:** COpAT, monitoring parameters, OPAT, outpatient parenteral antimicrobial therapy, readmission factors

## Abstract

Outpatient parenteral antimicrobial therapy (OPAT) has become more common in infectious diseases practice settings. Similarly, OPAT-related publications have also increased. The objective of this article was to summarize clinically important OPAT-related publications from 2023. Eighty-one articles were found on initial search, with 52 meeting inclusion criteria. A survey containing the 19 articles that had at least 1 citation was sent to an email listserv of multidisciplinary clinicians with OPAT experience. This article summarizes the “top 10” 2023 OPAT articles from the survey results.

## Teaser

As OPAT utilization increases, so does its associated research. Staying abreast of the emerging literature can be challenging. Herein a group of geographically and clinically diverse OPAT practitioners summarize the “top 10” OPAT papers from 2023 to ease this burden

Outpatient parenteral antimicrobial therapy (OPAT) is defined as the receipt of ≥2 doses of parenteral antimicrobials outside of the acute care setting [1]. OPAT is part of a broader concept of complex outpatient antimicrobial therapy (COpAT), which includes both oral and intravenous (IV) antimicrobials given for a prolonged duration and usually requires close monitoring [2]. OPAT and COpAT have grown to incorporate more time and resources in infectious diseases (ID) practice, and correspondingly, publications in the area of OPAT have also grown. In this article, we summarize 10 important OPAT publications from 2023 as voted on by a national multidisciplinary group of OPAT clinicians.

## METHODS

**Figure 1.**
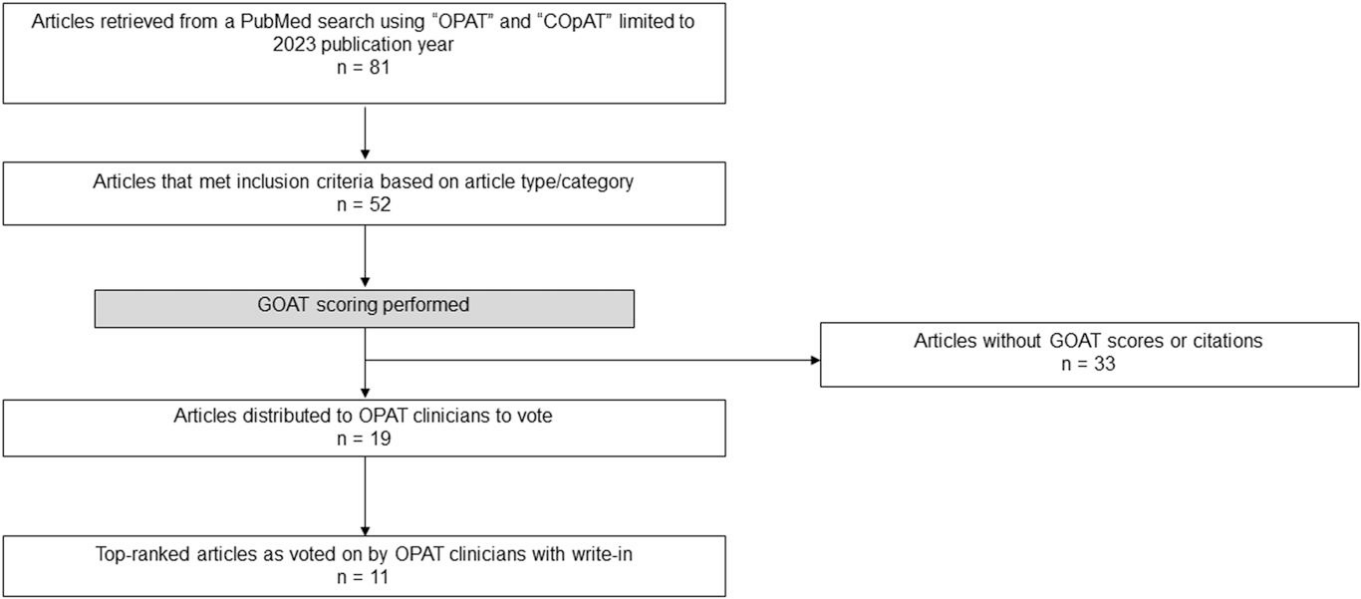
Selection of articles. Abbreviations: COpAT, complex outpatient antimicrobial therapy; GOAT, Grading Outcomes–based research in Antimicrobial Therapy; OPAT, outpatient parenteral antimicrobial therapy.

A Medline search was performed using the key terms “OPAT” and “COpAT” to identify PubMed-indexed publications with a citation date between 1 January and 31 December 2023. Identified articles were then reviewed to ensure publication in 2023; articles published (including electronic publication) before 2023 were excluded, as were narrative reviews without new data, opinion pieces, and in vitro–only studies. Of the 81 publications identified from the Medline search, 52 met inclusion criteria and were assigned a Grading Outcomes–based research in Antimicrobial Therapy (GOAT) score [3, 4] on the same day, minimizing the chance of score fluctuation. In brief, the GOAT score considers the publishing journal's impact factor, the article's number of citations, and time between publication and date the GOAT score was calculated [3, 4]. Articles without a GOAT score or any citations were excluded from further consideration. Therefore, the 14 remaining articles with GOAT scores were included in a survey. Five additional articles were published in journals without an impact factor but were cited by other manuscripts; therefore, the authors decided to include these articles in the survey given a relatively limited number of OPAT articles with GOAT scores. See Figure 1 for the article selection process.

A survey containing 19 studies was created, with the articles in alphabetical order by first author's last name. This was sent to a national email listserv of 289 clinicians who expressed interest and practice experience in OPAT to vote for the top 10 OPAT articles of 2023. The survey recipients were blinded to the GOAT scores (except for the 3 authors who calculated the scores and built the survey). When selecting their top 10 articles, OPAT clinicians were asked to consider clinical practice applicability, feasibility, and innovation. There was also an option for a write-in article. Eighty-eight survey responses were received (30% response rate). There were multiple votes for 1 write-in article, therefore an “honorable mention” was included. The final list was distributed to have each author summarize 1–2 articles, avoiding any that they may have co-authored. The 11 articles are presented alphabetically by the first author's last name below. In Table 1, the articles are divided into (1) programmatic studies, those that looked at the impact of OPAT programs on different outcomes; and (2) therapy-based studies, which compared outcomes between therapies [5–15].

**Table 1.** Summary of Top Publications on Outpatient Parenteral Antimicrobial Therapy From 2023

Citation	Study Design	Primary and Secondary Outcomes	Strengths/Limitations/Potential Impacts
Programmatic studies			
Agnihotri et al [5]	Retrospective, quasi-experimental study of patients who received OPAT either before or after implementation of an OPAT care team (ID physician, OPAT RN)	Primary outcome: Unplanned OPAT-related readmissions decreased after implementation of the OPAT team (17.8% vs 7%) Secondary outcomes Decrease in all-cause readmissions not significant (26% vs 18.9%) Clinical cure rates improved with OPAT team care (69.8% vs 94.9%)	Strengths Real-life Limitations Single-center study, may limit generalizability Retrospective design may not capture all events Potential impacts A structured multidisciplinary OPAT program can decrease readmissions and clinical cure
Billmeyer et al [6]	Retrospective cohort study to describe the frequency and risk factors associated with OPAT AEs and unscheduled care	Primary outcome Any ADE: 30.9% Risk factors: Receipt of lipo/glycopeptide (OR, 5.14 [95% CI, 2.47–10.99]) VAD complication: 21.5% Risk factors: Obesity (OR, 3.32 [95% CI, 1.38–8.73]), multidrug therapy (OR, 2.56 [95% CI, 1.21–5.39]) OPAT-related ED visits: 21.9% Risk factors: Any ADE (OR, 2.19 [95% CI, 1.13–4.22]), VAD complication (OR, 2.37 [95% CI, 1.15–4.86])	Strengths Identified potentially modifiable risk factors Limitations Single-center study, may limit generalizability Retrospective design may not capture all events Potential impacts Addition of an ID-trained pharmacist in OPAT care may reduce OPAT-related AEs
Douglass et al [8]	Retrospective cohort study, reviewing RN care coordination in 53 episodes of dalbavancin administration in PWUD	Primary outcomes 100% of dalbavancin courses were completed RN interventions were needed in 32 of 53 episodes Patients with outpatient dalbavancin administration had 143 total RN interventions Prospectively, RN time spent for 7 patients, over 1 mo, was 179 min in care coordination	Strengths Real-world demonstration of RN time spent in care coordination for successful dalbavancin treatment in PWUD Limitations Single-center, retrospective study Time-tracking in small number of patients Potential impacts Dalbavancin use is associated with significant care coordination time and effort
Drew et al [9]	Retrospective cohort study utilizing an EHR-available prediction model for 30-d readmission rates	Primary outcomes 30-d readmissions occurred in 94 (20.1%) patients, of which 56 (12%) were OPAT-related Neither maximum (uOR, 1.00 [95% CI, .98–1.03]) nor discharge (uOR, 1.01 [95% CI, .98–1.03]) scores predicted readmissions Adjusting for patient age at index admission, vancomycin use, IV drug use, or site of OPAT receipt improved the prediction models	Strengths First study to incorporate this readmission prediction score to the OPAT population Limitations Single-center, retrospective study Only applicable to this specific EHR and the variables used Prediction scores were only selected once per day, rather than every 4 h as with the live, inpatient version updates Potential impacts More study is needed to identify predictor factors for OPAT readmission
Frisby et al [11]	Retrospective cohort of patients receiving OPAT to determine the relevance of different laboratory tests for OPAT follow-up and complications	Primary outcomes OPAT complications associated with: CCI score >5 (aOR, 2.21 [95% CI, 1.11–4.43]) OPAT duration >5 wk (aOR, 2.7 [95% CI, 1.3–5.6]) Hospital readmissions associated with: Abnormal CMP (aOR, 5.7 [95% CI, 2.85–11.24])	Strengths Robust set of variables evaluated Limitations Single-center study, may limit generalizability Retrospective design may not capture all events Potential impacts More study is needed to determine ideal laboratory monitoring guidance
Kaul et al [12]	Retrospective cohort study investigating factors associated with OPAT loss to follow-up	Primary outcome: Risk factors for OPAT loss to follow-up: Discharge to SAR (OR, 3.24 [95% CI, 2.35–4.47]) Discharge to LTCF (OR, 5.91 [95% CI, 2.89–12.03]) Federal insurance status (OR, 1.493 [95% CI, 1.04–2.14])	Strengths Robust set of variables evaluated, large patient population, long-term data access First study to investigate factors related to loss to follow-up Limitations Single-center study, may limit generalizability Retrospective design may not capture all events Potential impacts: Additional care coordination efforts may be needed for patients with federal insurance and those who are discharged to a SAR or LTCF
Lam et al [13]	Retrospective cohort of OPAT patients followed by an OPAT clinic in Canada	Primary outcome: Of 2513 OPAT courses, there were 55 cases of neutropenia, resulting in an incidence of 2.2 cases per 100 treatment courses (95% CI, 1.7–2.9) Secondary outcomes Vancomycin (21/541 [3.9%]), ceftriaxone (10/490 [2.0%]), and cloxacillin (2/103 [1.9%]) were the most common causative agents of neutropenia Five (9.1%) of patients with neutropenia were admitted All patients recovered neutrophils and there were no related deaths	Strengths Large cohort of OPAT patients studied over multiple years Specific to the OPAT setting Practical study question that informs the UK and IDSA OPAT guideline laboratory monitoring recommendations Limitations Variability of OPAT monitoring frequency depending on receipt of vancomycin or not, which may have impacted the time to neutropenia detection No information provided on antimicrobial dosing or methods of infusion No data on high-risk oral monotherapy such as linezolid or sulfamethoxazole-trimethoprim Potential impacts Monitoring for neutropenia should occur at least at week 3 of treatment
Therapy-based studies			
Broermann et al [7]	Retrospective cohort study of patients being treated for uncomplicated streptococcal BSI with either standard course of IV therapy or oral therapy 3–9 d after initial IV therapy	Primary outcome: Treatment failure in 12% of IV-only therapy vs 4.4% of partial PO therapy (HR, 0.53 [95% CI, .18–1.60]) Secondary outcome: Partial PO therapy group was associated with shorter length of stay (−2.23 d [95% CI, −3.53 to −.94])	Strengths Provides a time frame for transition from IV to oral therapy in streptococcal BSI Use of propensity score analysis and early clinical failure criteria to adjust for therapy response at time of transition from oral to IV therapy Limitations Small sample size from 1 hospital system Retrospective design may limit generalizability and introduce bias of patients selected with favorable prognoses Potential impacts Transitioning to PO therapy may provide for shorter length of hospital stay
Frazier et al [10]	Retrospective cohort study comparing readmission rates between SOC and dalbavancin therapy for SAB	Primary outcome: Treatment-related readmission within 30 d: 22% SOC vs 15% dalbavancin (*P* = .484) Secondary outcomes Treatment-related readmissions within 90 d: 22% SOC vs 19% dalbavancin (*P* = .735) Regimen adherence: 44% SOC vs 85% dalbavancin (*P* < .001)	Strengths Retrospective study design and single center Represented a vulnerable population Limitations Did not utilize standardized dalbavancin dosing Did not assess duration of therapy Potential impacts Dalbavancin is an option for SAB in patients with barriers to care
Shi et al [14]	Retrospective review of adult patients who received vancomycin via OPAT and had both trough and AUC levels available	Primary outcomes Discordance in vancomycin dose adjustment prompted by 24-h AUC compared to strict trough (15–20 mg/L) or relaxed (10–20 mg/L) goals Discordance occurred in 51% of patients in strict vs 16% of patients in relaxed trough goal categories compared to AUC dosing	Strengths Patients served as their own controls Largest cohort of OPAT AUC- vs trough-based comparison Compared various trough goals, mimicking real-world practice Limitations Single center and retrospective May not apply to 2-level or other AUC calculations Only safety and not efficacy was evaluated Potential impacts Vancomycin troughs between 12 and 16 mg/L are likely in goal AUC range Selective AUC monitoring when troughs are <12 or >16 are reasonable
Sunagawa et al [15]	Retrospective review of ADRs experienced by patients receiving cefazolin or ceftriaxone in OPAT	Primary outcome: Clinically significant AEs: 2.7 per 1000 set of weekly labs Secondary outcomes Time to AEs: median, 17 d (IQR, 10–27 d) Unplanned healthcare utilization: 14.1%	Strengths Standardized definitions for AEs High percentage of weekly safety labs obtained Limitations Single-center study, may limit generalizability Retrospective design may not capture all events Presumed causation of laboratory abnormalities to AEs Potential impacts Lab monitoring for cefazolin and ceftriaxone could be less frequent than weekly

Abbreviations: ADE, adverse drug event; ADR, adverse drug reaction; AE, adverse event; aOR, adjusted odds ratio; AUC, area under the curve; BSI, bloodstream infection; CCI, Charlson Comorbidity Index; CI, confidence interval; CMP, complete metabolic profile; ED, emergency department; EHR, electronic health record; HR, hazard ratio; ID, infectious diseases; IDSA, Infectious Diseases Society of America; IQR, interquartile range; IV, intravenous; LTCF, long-term care facility; OPAT, outpatient parenteral antimicrobial therapy; OR, odds ratio; PO, oral; PWUD, persons who use drugs; RN, registered nurse; SAB, *Staphylococcus aureus* bacteremia; SAR, subacute rehabilitation facility; SOC, standard of care; UK, United Kingdom; uOR, unadjusted odds ratio; VAD, vascular access device.

## PUBLICATION SUMMARIES

### Decreased Hospital Readmissions After Programmatic Strengthening of an Outpatient Parenteral Antimicrobial Therapy (OPAT) Program

In this retrospective, quasi-experimental study, Agnihotri et al [5] examined hospital readmission rates both before and after the implementation of a dedicated OPAT team, composed of a full-time registered nurse (RN) and a 0.1 full-time equivalent physician. The primary endpoint of the study was unplanned OPAT-related admissions during the time of the OPAT course. Secondary outcomes included all-cause hospital readmissions during the OPAT course and successful versus failed treatment outcome.

The study evaluated 428 patients: 73 in the preintervention arm and 355 in the postintervention arm. The OPAT-related readmission rate decreased from 17.8% to 7% (*P* = .003). Reasons for OPAT-related admission included infection recurrence or progression, adverse drug reaction, and line-associated issues. The decrease in all-cause readmissions was not statistically significant (18.9% vs 26%; *P* = .165). For those patients in whom clinical cure could be evaluated, there was a significantly higher cure rate in the postintervention arm (94.9% vs 69.8%; *P* < .001).

The findings from this study lend support to having a structured, multidisciplinary OPAT program, as the rates of OPAT-related readmission and clinical cure improved after implementation of such a program.

### Predictors of Adverse Safety Events and Unscheduled Care Among an Outpatient Parenteral Antimicrobial Therapy (OPAT) Patient Cohort

While there are several studies evaluating predictors of adverse events (AEs) and unscheduled OPAT-related medical care for patients, data incorporating an ID pharmacist into this process are mixed. Billmeyer et al [6] looked to evaluate predictors of adverse safety events in OPAT patients within a single institution's home-based OPAT process. The study time period included a collaborative OPAT program, allowing an ID pharmacist to review admitted patients for potential OPAT, antibiotic reconciliation and optimization, identification of a designated OPAT provider, and recommending additional monitoring.

Over a 30-month study period, 265 patients were included for evaluation. Eighty-two (30.9%) patients experienced an adverse drug event (ADE), 30 (11.3%) of which were defined as severe or serious, where “severe” was defined as requiring significant intervention and “serious” was defined as resulting in hospitalization, death, a life-threatening condition, significant disability, or birth defect. Risk factors for serious ADEs included Black or of African-American race, longer planned durations, and/or receipt of a lipo/glycopeptide (vancomycin or daptomycin). Meanwhile, bloodstream infections (BSIs) or an encounter with the ID pharmacist were associated with decreased odds of a serious ADE. OPAT-related emergency department (ED) visits (OREDVs) occurred in 21.9% of patients. Patients were more likely to have an OREDV if they had an ADE or vascular access complication and were less likely to have an OREDV if diagnosed with skin infections or endocarditis. Like OREDV, 90-day hospital admissions occurred somewhat frequently in patients (20.0%). While ADE remained significant in the multivariable predictor model, vascular access complications did not.

Adverse drug events remain a persistent predictor of both OPAT-related ED visits and hospital readmissions. Addition of an ID-trained pharmacist into OPAT may decrease ADEs.

### Intravenous Versus Partial Oral Antibiotic Therapy in the Treatment of Uncomplicated Bloodstream Infection due to *Streptococcus* Species

Bloodstream infections secondary to β-hemolytic streptococci rank among the top pathogens responsible for BSIs and have a reported 30-day case fatality rate of 11% [16, 17]. Thus, optimizing antibiotic therapy is key to treatment and ultimately reducing morbidity and mortality. While standard therapy has traditionally included only parenteral antibiotics, utilization of oral antibiotics in combination with parenteral antibiotics has increased given convenience and high bioavailability of some oral antibiotics. Broermann et al [7] published a retrospective cohort study to describe effectiveness of partial oral antibiotic therapy regimens in patients with uncomplicated β-hemolytic streptococci BSIs.

Standard IV therapy was defined as receiving IV antibiotics for a minimum of 7 days. Partial therapy was defined as a transition from IV to oral antibiotic therapy 3 to 9 days after BSI and completing a minimum of 4 days of oral antibiotic therapy. Patients with complicated BSIs or those whose antibiotic regimens did not meet the definitions as described were excluded. Ultimately, 222 patients were included in the study: 99 receiving standard IV therapy and 123 in the oral therapy group. The most commonly isolated pathogen was β-hemolytic streptococci (87 [39%]) followed by *Streptococcus pneumoniae* (76 [34%]). Median antibiotic duration in both groups was 14 days, with 4 days being the median duration of IV antibiotics in the oral antibiotic group. Beta-lactams (62 [50%]) were the most commonly used antibiotics, followed by fluoroquinolones (47 [38%]).

Treatment failure rates were 4.4% in the oral antibiotic therapy group, compared to 12% in the standard IV therapy group, which, after adjustments for confounding variables and propensity to receive oral therapy in a multivariate Cox model, suggested no difference between the 2 groups. Identified risk factors for treatment failure included older age, a cancer diagnosis, and skilled nursing facility residency. The partial oral antibiotic therapy group was associated with shorter hospital length of stay (2.23 days fewer) after adjustments were made for propensity to receive oral antibiotic therapy and other confounders in the multivariate model.

While this study does have a small sample size and is from a single hospital system, the risk stratification of eligible patients and timeline of transition from IV to oral antibiotics provides clinicians with valuable data, potentially decreasing length of hospital stay and complications associated with longer-term OPAT.

### The Hidden Cost of Dalbavancin: OPAT RN Time Required in Coordination for Persons Who Use Drugs

Dalbavancin is a long-acting lipoglycopeptide with convenient once-weekly dosing that has been used to treat serious infections in persons who use drugs (PWUD) [18, 19]. However, there is additional care coordination needed to ensure successful medication administration for treatment success. Douglass et al [8] described a retrospective cohort study of RN time required for coordination of care for PWUD in 53 dalbavancin courses (in 52 patients) administered for severe infection. Substance use was identified by *International Classification of Diseases, Tenth Revision* or chart documentation and included any history of active/prior substance use. Infections included 34 bone and joint infections, 5 endocarditis, 7 BSI alone, 5 skin/soft tissue infection, and others. The study included 27 with IV opioid use and 27 with IV methamphetamine use. The most common reasons for dalbavancin use included prior IV drug use (36), unsafe home situation (13), and prior nonadherence to medications (10). Dalbavancin was administered inpatient in 31 patients, via infusion center (14), via home infusion (11), and other site (3); 100% of courses were completed. Weekly dalbavancin doses varied from 500 to 1500 mg. Interventions were needed for 32 of 53 regimens. For outpatient doses, 17 needed RN intervention. Additionally, a separate prospective review of RN time spent over 1 month providing outpatient coordination for 7 patients demonstrated 19 interventions and 179 minutes of care coordination (mean, 8.9 minutes per episode). Common tasks performed included patient care coordination (16), coordination with vendor (14), confirming dose administration (12), entering orders (10), and transitional care management call (10). One AE occurred, and 2 recurrences/relapses of infection requiring readmission occurred. Despite its convenient dosing, successful dalbavancin administration in PWUD can require significant care coordination efforts.

### Electronic Health Record–Based Readmission Risk Model Performance for Patients Undergoing Outpatient Parenteral Antibiotic Therapy (OPAT)

Upward of 25% of OPAT patients may experience unplanned 30-day hospital readmissions [20]. Low-effort, high-impact interventions aimed at reducing this number are needed. This retrospective study by Drew et al investigated the use of an electronic health record–embedded automated 30-day readmission prediction model readily available to inpatient teams [9]. Variables in the model include patient age, clinical diagnosis, laboratory values, medication numbers and classes, order types, and healthcare utilization variables. While the model updated every 4 hours while inpatient, the authors selected 1 daily score and evaluated the score on discharge day and overall maximum score. Additionally, the authors evaluated whether patient age at index admission, vancomycin use, IV drug use, and site of OPAT care impacted the model score. A total of 467 unique patient encounters were included, with 94 unplanned readmissions; of those, 56 were OPAT related. Using univariable logistic regression, neither the maximum model score, nor the discharge model score, was associated with 30-day unplanned readmission (unadjusted odds ratio, 1.00 [95% confidence interval {CI}, .98–1.03] and 1.01 [95% CI, .98–1.03], respectively). Neither multivariable logistic regression nor incorporation of the additional OPAT variables improved the predictability of the score. The authors concluded that additional research should be performed to determine the unique OPAT patient variables that predict unplanned readmission rates and were not captured in this prediction model.

### Dalbavancin Versus Standard of Care for *Staphylococcus aureus* Bacteraemia in Patients Unable to Receive Outpatient Parenteral Antimicrobial Therapy

Frazier et al [10] performed a single-center study of adults treated by the ID consult service at a large hospital in the southeastern United States during 1 January 2016–31 August 2021 for monomicrobial *Staphylococcus aureus* bacteremia (SAB) with either at least 1 dose of dalbavancin or at least 7 days of standard-of-care antibiotic therapy (SOC; defined as cefazolin or nafcillin for methicillin-susceptible *S aureus* and vancomycin or daptomycin for methicillin-resistant *S aureus*) at discharge. Patients with a creatinine clearance <30 mL/min or who were transferred to external nursing facilities were excluded. The primary outcome was 30-day treatment-related readmission.

Fifty-four patients (n = 27 in each arm at discharge) were included, among whom patients treated with dalbavancin were younger (median, 42 vs 52 years; *P* = .039), more often had documented histories of noncompliance to therapy (55% vs 6%; *P* < .001), and primarily received a 2-dose series of dalbavancin (78%). Overall, 75% of subjects had a prespecified barrier to care, most often including historic or active substance use disorder, lack of insurance, or history of noncompliance. Most patients in both arms had a primary bacteremia; rates of metastatic complications were similar, except that all 5 cases of epidural abscess were treated with SOC. Readmission rates with dalbavancin versus SOC were similar at both 30 days (15% vs 22%; *P* > .05) and 90 days (19% vs 22%; *P* > .05). Adherence was described as being higher with dalbavancin (85% vs 44%), with the caveat that nonadherence was not clearly defined, appearing to include anyone receiving <100% of the originally prescribed regimen, and so may not be clinically meaningful.

These results should be considered cautiously and in the context of prior studies challenging the notion that people who inject drugs cannot receive standard daily OPAT [21] as well as multiple studies and clinical practice guidance indicating that oral antibiotics can be an effective alternative to daily IV therapy for patients with serious injection-related infections, including SAB [22–24]. Additionally, an already concluded randomized controlled trial [25] (NCT04775953) should soon establish whether dalbavancin is as efficacious as standard daily IV antimicrobials for complicated SAB. In general, this study suggests that dalbavancin can be part of the therapeutic armamentarium for vulnerable patients with barriers to daily OPAT.

### Usefulness of Routine Laboratory Tests for Follow-up of Patients Receiving Outpatient Parenteral Antimicrobial Therapy Run by Infectious Diseases Fellows

The Infectious Diseases Society of America (IDSA) OPAT guidelines recommend serial laboratory testing in patients receiving OPAT [1]. However due to a lack of data, the guidelines did not make recommendations for specific tests and laboratory monitoring frequency.

Frisby et al [11] attempted to address this lack of data. They performed a retrospective cohort study over 7 years to measure risk factors for abnormal laboratory tests and determine the impact of abnormal laboratory tests on OPAT-related complications including need for antibiotic change or hospital readmission.

In their analysis, 246 of 326 total patients were considered eligible. Of these 246 patients, 116 had OPAT-related complications. A Charlson Comorbidity Index [26] score of <5, the use of ceftriaxone, and OPAT durations of 2–4 weeks were associated with fewer OPAT-related complications.

The majority of laboratory abnormalities included abnormal vancomycin troughs, creatinine elevations, or electrolyte abnormalities, suggesting that these tests were of higher value. There were also 11 incidences of elevated liver enzymes. Abnormal comprehensive metabolic panels were associated with hospitalization or changing antibiotics, while other laboratory abnormalities were not associated with a need for hospitalization or change in antibiotics, suggesting that comprehensive metabolic panels were of higher value. In addition, a larger proportion of patients in the second week of OPAT had abnormal laboratory test results and required a hospitalization or change in antibiotic, suggesting that this time period may be higher yield for laboratory testing. It is unclear why an OPAT duration of 2–4 weeks was associated with fewer OPAT-related complications while abnormal laboratory test results in the second week were both more likely to occur and to lead to readmissions. Unorthodox methodological choices in the building of the multivariate models may have contributed to these disparate results. While this study contributed to the breadth of knowledge, the optimal timing of laboratory monitoring in OPAT patients is still unknown.

### Factors Associated With Loss to Follow-up in Outpatient Parenteral Antimicrobial Therapy: A Retrospective Cohort Study

There have been many studies looking at factors associated with readmission rates in OPAT patients [27, 28], but what factors are associated with loss to follow-up is less clear.

In this retrospective cohort study, Kaul et al [12] sought to determine which factors led to an increased risk of loss to follow-up with ID staff in patients receiving OPAT. The study took place at 4 hospitals within 1 urban health system, and patients were discharged to either home, subacute rehabilitation facility (SAR), acute rehabilitation facility, or long-term care facility (LTCF). Of the 1528 OPAT patients who were recommended to have ID follow-up, only 1110 (72.6%) were seen in follow-up, resulting in a loss to follow-up rate of 27.4%. The factors found to be associated with loss to follow-up included discharge to a SAR or LTCF or having federal insurance. No other socioeconomic or demographic factors were found to be associated with loss to follow-up. Reasons for the loss to follow-up for those discharging to a SAR or LTCF are unclear, but might include the challenges of care coordination between OPAT programs in a healthcare system and facilities outside of their system.

### Antibiotic-Induced Neutropenia in Patients Receiving Outpatient Parenteral Antibiotic Therapy: A Retrospective Cohort Study

While the United Kingdom and IDSA OPAT guidelines recommend at least once-weekly laboratory monitoring for all OPAT patients given the risks of antimicrobial-related adverse effects, the evidence behind this recommendation is limited [1, 29].

In this single-center retrospective cohort study, Lam et al [13] describe the incidence and management of antibiotic-induced neutropenia over a 7-year period. Patients receiving vancomycin had weekly complete blood count (CBC) with differential and those on other antibiotics received CBC with differential starting at week 4 of therapy. Neutropenia was defined as an absolute neutrophil count of ≤1.5 × 10^9^/L and confirmed by manual chart review. The incidence of neutropenia per 100 treatment courses was determined for each antimicrobial. The median time to neutropenia and median neutrophil count at the time of diagnosis was assessed for the cohort.

Of 2513 OPAT courses, 55 cases of antibiotic-induced neutropenia were identified (15 mild, 22 moderate, and 16 severe), providing an incidence of 2.2 cases per 100 treatment courses (95% CI, 1.7–2.9). The median duration of antibiotic therapy was 6 weeks. The median time to neutropenia diagnosis was 25 (interquartile range, 19–30) days. Vancomycin (21/541 [3.9%]), ceftriaxone (10/490 [2.0%]), and cloxacillin (2/103 [1.9%]) were the most common causative agents. A minority of neutropenic patients had new symptoms (27.2%), all patients recovered neutrophil counts after discontinuation/completion of therapy, and there were no related deaths. Five (9.1%) symptomatic patients required hospital admission. In 9 cases (16.3%), a β-lactam antibiotic was changed to another β-lactam with a different side chain, and all successfully recovered neutrophils.

The authors conclude that with standardized outpatient monitoring during the third week of OPAT, cases of neutropenia can be detected and managed as outpatients. This study adds to the growing body of literature that informs OPAT laboratory monitoring frequency. However, it is possible that neutropenia cases may have been detected earlier for non-vancomycin antimicrobials if monitoring routinely commenced before week 3.

### In Outpatients Receiving Parenteral Vancomycin, Dosing Adjustments Produced by Area Under the Curve–Based and Trough-Based Monitoring Differ Only at the Extremes of the Therapeutic Trough Range

Much has been debated regarding the optimal dosing and monitoring of vancomycin, including in the OPAT population [30, 31]. Many institutions utilize 1- or 2-level area under the curve (AUC) dosing while inpatient, but struggle with the optimal use of resources once the patient is discharged. Shi et al, at an academic medical center, retrospectively reviewed data from 115 patients who received vancomycin during OPAT and had both AUC- and trough-based monitoring performed [14]. AUC calculations were performed via Bayesian modeling, with a goal of 400–600 mg × hour/L, and trough goals were evaluated with strict goals of 15–20 mg/L and more relaxed goals of 10–20 mg/L.

Discordance occurred in 51% of the cases when strict trough goals were compared to AUC-based recommendations. However, discordance decreased to 16% when comparing AUC-based to the relaxed trough goals. Additionally, the authors found that agreement was near perfect (97.6%) when trough levels were 12–16 mg/L. Otherwise, AUCs were commonly below goal when troughs were <12 mg/L and above goal when troughs were >16 mg/L. Acute kidney injury only occurred in 8 (7%) patients, with only 1 patient requiring a change to alternative therapy and none resulting in readmission or an ED visit. Factoring in the time required for their OPAT pharmacists to evaluate, recommend changes, and document activities, the authors suggested that selective AUC calculation (eg, in patients with trough levels <12 or >16 mg/L) could offer similar safety profiles but significantly decrease workload.

### Honorable Mention: Serious Adverse Events and Laboratory Monitoring Regimens for Outpatient Parenteral Antimicrobial Therapy With Cefazolin and Ceftriaxone

The optimal frequency of OPAT laboratory monitoring is often stated to be weekly, according to the IDSA OPAT guidelines [1]. However, some IV antibiotics are likely better tolerated and could potentially be monitored less frequently. Sunagawa et al [15] investigated the AE rates of OPAT patients receiving cefazolin or ceftriaxone to try to determine if less frequent monitoring is needed.

Investigators reviewed OPAT courses for patients receiving cefazolin or ceftriaxone between 1 March 2019 and 30 September 2022. Patients who received concomitant IV antibiotics were excluded, but patients receiving concomitant oral antibiotics were included. The primary outcome was clinically significant OPAT AEs, which were defined as antimicrobial-associated AEs that led to a change in antimicrobial therapy or any line-associated AEs that needed intervention. Secondary outcomes included time on antimicrobial therapy until clinically significant AEs, unplanned healthcare utilization, and OPAT team actions undertaken to obtain or clarify laboratory data.

The study included 708 OPAT courses, about half receiving cefazolin and half receiving ceftriaxone. Fifty-five clinically significant OPAT AEs were found (7.8%), with 20 (2.8%) of them being drug-associated and 35 (5%) of them being line-associated. Only 9 of the drug-associated OPAT AEs were recognized via laboratory monitoring, and only 3 required unplanned healthcare utilization. The median duration of therapy before drug-associated OPAT AEs was 31 days. The incidence of laboratory monitoring–detected clinically significant AEs was found to be 2.7 per 1000 sets of weekly labs. The median duration of therapy before line-associated OPAT AEs was 16 days. There were no significant differences between those with or without clinically significant drug-associated OPAT AEs in relation to antibiotic used, duration of therapy, or OPAT modality. Patients receiving OPAT via hemodialysis lines or peripherally inserted central catheters were less likely to have a line-related AE than those with a midline. The authors suggest that, based on these results, laboratory monitoring of cefazolin and ceftriaxone OPAT regimens could be less frequent than once weekly.

## CONCLUSIONS

Our multidisciplinary group of OPAT clinicians summarized the “top 10” OPAT publications from 2023, as voted on by a national multidisciplinary group of OPAT clinicians. Common themes that emerged from the top 10 articles included OPAT AEs and readmission factors, dalbavancin use, and OPAT team monitoring approaches. Compared with the 2022 top 10, factors for OPAT AEs and readmission remained a common theme. Based on the 2023 top 10 articles, remaining literature gaps include identifying a reliable predictor model for OPAT-related readmissions that can be used before hospital discharge and determining the ideal laboratory monitoring frequency for OPAT programs based on antimicrobial regimen.

## Notes

***Author contributions*.** Study design: All authors. Literature search: L. M. C.-K. and K. L. R. Manuscript scoring: L. M. C.-K., K. L. R., and M. V. M. Voting for top 10 articles: All authors. Table and figure creation: L. M. C.-K. and M. V. M. Manuscript preparation and review: All authors.

***Patient consent*.** This work does not include factors necessitating patient consent.
